# Increased Protein Stability of CDKN1C Causes a Gain-of-Function Phenotype in Patients with IMAGe Syndrome

**DOI:** 10.1371/journal.pone.0075137

**Published:** 2013-09-30

**Authors:** Naoki Hamajima, Yoshikazu Johmura, Satoshi Suzuki, Makoto Nakanishi, Shinji Saitoh

**Affiliations:** 1 Department of Pediatrics, Nagoya City West Medical Center, Nagoya, Aichi, Japan; 2 Department of Cell Biology, Nagoya City University Graduate School of Medical Sciences, Nagoya, Aichi, Japan; 3 Department of Pediatrics and Neonatology, Nagoya City University Graduate School of Medical Sciences, Nagoya, Aichi, Japan; Baylor College of Medicine, United States of America

## Abstract

Mutations in the proliferating cell nuclear antigen (PCNA)-binding domain of the *CDKN1C* gene were recently identified in patients with IMAGe syndrome. However, loss of PCNA binding and suppression of CDKN1C monoubiquitination by IMAGe-associated mutations hardly explain the reduced-growth phenotype characteristic of IMAGe syndrome. We demonstrate here that IMAGe-associated mutations in the *CDKN1C* gene dramatically increased the protein stability. We identified a novel heterozygous mutation, c.815T>G (p.Ile272Ser), in the *CDKN1C* gene in three siblings manifesting clinical symptoms associated with IMAGe syndrome and their mother (unaffected carrier). PCNA binding to CDKN1C was disrupted in the case of p.Ile272Ser, and for two other IMAGe-associated mutations, p.Asp274Asn and p.Phe276Val. Intriguingly, the IMAGe-associated mutant CDKN1C proteins were fairly stable even in the presence of cycloheximide, whereas the wild-type protein was almost completely degraded via the proteasome pathway, as shown by the lack of degradation with addition of a proteasome inhibitor, MG132. These results thus suggested that the reduced-growth phenotype of IMAGe syndrome derives from CDKN1C gain-of-function due to IMAGe-associated mutations driving increased protein stability.

## Introduction

IMAGe syndrome (OMIM 614732) was originally defined as an association of intrauterine growth restriction, metaphyseal dysplasia, adrenal hypoplasia congenita, and genital anomalies [1]. A number of familial and sporadic cases, which show clinical heterogeneity, have been reported [1]–[8]. The genetic cause of this syndrome has recently been shown to be mutations in the proliferating cell nuclear antigen (PCNA)-binding domain of the *CDKN1C* gene [9].

**Figure 1:**
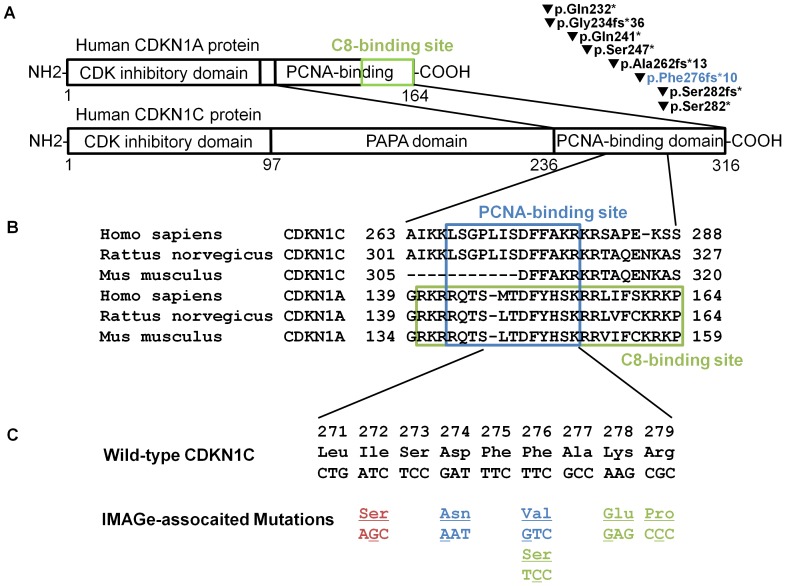
Structure of CDKN1C and CDKN1A proteins and IMAGe-associated mutations. (A) Schematic representation for the structures of human CDKN1C and CDKN1A proteins and for the BWS-associated truncation mutations in the PCNA-binding domain of CDKN1C. The green closed square represents the C8-binding site [13]. Numbers below the schemas represent the locations of amino acid residues. Filled inverted triangles denote the truncation mutants in the PCNA-binding domain of CDKN1C reported in patients with BWS [11], [12]. The blue characters represent the mutation analyzed in this article (p.Phe276fs*10). (B) Alignment of amino acid sequences around the PCNA- and C8-binding sites in human, rat, and mouse CDKN1C and CDKN1A. The numbers above the set of sequences represent the amino acid residues. The blue closed square represents the PCNA-binding site [17] and the green closed square represents the C8-binding site [13]. Multiple sequence alignment was performed by using ClustalW (http://www.genome.jp/tools/clustalw/). Accession numbers of the amino acid sequences described here are as follows: NP_000067.1, Homo sapiens CDKN1C; NP_001028929.1, Rattus norvegicus CDKN1C; NP_001155096.1, Mus musculus CDKN1C; AAH13967, H. sapiens CDKN1A; AAI00621, R. norvegicus CDKN1A; and AAH02043, M. musculus CDKN1A. (C) Amino acid and nucleotide sequences of the PCNA-binding domain in the wild-type and IMAGE-associated mutant *CDKN1C* genes in human. Numbers on the top line represent amino residues of the CDKN1C protein based on accession number NM_000076.2. Red characters represent the mutation reported in this article (c.815T>G and p.Ile272Ser). Blue and green characters represent mutations described in the previous report [9]: blue characters represent mutations analyzed in this article (p.Asp274Asn and p.Phe276Val). Underlined characters represent substituted residues and nucleotides.

CDKN1C (p57Kip2), CDKN1A (p21Cip1), and CDKN1B (p27Kip1) belong to the Cip/Kip family of cyclin-dependent kinase (CDK) inhibitors (Figure 1A), which negatively regulate cell cycle progression by inhibiting G1 CDKs [10], [11]. The *CDKN1C* gene is located at 11p11.5, which harbors a cluster of imprinted genes and is expressed only from the maternal allele. Mutations across the length of the *CDKN1C* gene have been identified in patients with Beckwith-Wiedemann syndrome (BWS), which is characterized by an over-growth phenotype and an association with certain cancers; loss-of-function of CDKN1C promotes cell proliferation giving rise to an over-growth phenotype [11], [12]. In contrast, the clinical symptoms of patients with IMAGe syndrome strongly suggest that mutations in their *CDKN1C* gene are associated with gain-of-function of the CDKN1C protein, although disruption of PCNA binding and suppression of CDKN1C monoubiquitination do not directly correlate with the *CDKN1C* gain-of-function [9], and truncation mutants of CDKN1C lacking PCNA binding were also identified in BWS patients (Figure 1A) [11], [12].

In the present study, we identified a novel maternally inherited mutation in the PCNA-binding domain of the *CDKN1C* gene in three siblings manifesting symptoms associated with IMAGe syndrome. Molecular investigations demonstrated that the IMAGe-associated mutations caused a dramatic increase in the stability of the CDKN1C proteins that probably results in a functional gain.

## Subjects and Methods

### Subjects

**Figure 2:**
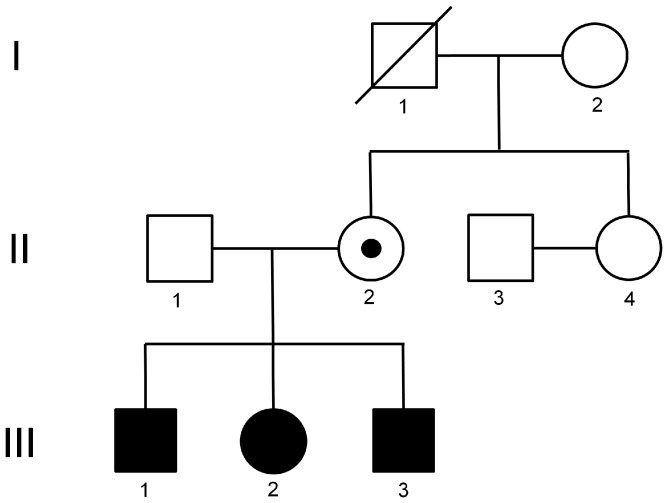
Pedigree of the family with IMAGe syndrome. Filled squares and circles represent the male and female patients, respectively. Closed squares and circles represent the male and female unaffected individuals, respectively. Small filled circles with large closed circles or squares represent unaffected carriers. Diagonal lines represent deceased individuals.

Three siblings, patient 1 (male, III-1 in Figure 2), 2 (female, III-2), and 3 (male, III-3), were born from non-consanguineous Japanese parents with normal adult heights (father (II-1), 182 cm; mother (II-2), 158 cm) and normal birth body weights and lengths. There is no other sibling in this family. The mother’s parents (I-1, I-2) and younger sister (II-4) were born with a normal body weight and length and are of normal adult height. All individuals other than the siblings in this family manifest no clinical symptoms associated with IMAGe syndrome. The siblings and their parents were subjected to molecular genetic analysis.

Clinical profiles of the siblings are summarized (Table 1). In brief, they presented with severe intrauterine growth restriction, frontal bossing, and a flattened nasal bridge. The males presented with hypospadias and bilateral cryptorchidism. All three siblings had experienced recurrent episodes of acute adrenal insufficiency, and their adrenals could not be detected by image analysis; their adrenal function is currently well managed by hydrocortisone replacement therapy. No metaphyseal dysplasia was observed. They presented with severe growth failure without growth hormone deficiency, and are undergoing growth hormone replacement therapy. Their psychomotor development is normal, although a vocal tic was observed in patient 2. No abnormal ophthalmologic findings including eyeball size were identified.

**Table 1:** Clinical profiles of three siblings with IMAGe syndrome.

	Patient 1 (male)	Patient 2 (female)	Patient 3 (male)
Present Age	15 years	12 years	10 years
Gestational Age	37 weeks 1 day	39 weeks 1 day	37 weeks 4 days
Body Weight at Birth	1374 g (−3.6 SD)	1772 g (−3.8 SD)	1808 g (−2.8 SD)
Body Height at Birth	38 cm (−4.5 SD)	41 cm (−4.4 SD)	41 cm (−3.2 SD)
Onset of Adrenal Insufficiency	4 months	1 month	8 days
Hydrocortisone Replacement Therapy	From 5 years	From 2 years	From 8 days
Growth Hormone ReplacementTherapy	From 11 years with height 105.7 cm (−5.5 SD)	From 8 years with height 102.1 cm(−4.5 SD)	From 6 years with height 95.7 cm(−3.7 SD)
Present Height	128.4 cm (−5.4 SD)	130.2 cm (−3.4 SD)	121.6 cm (−2.3 SD)
Metaphyseal Dysplasia	Not observed from 5 years	Not observed from 3 years	Not observed from 1 year
Other Bone Disease	Thin proximal phalanges,Perthes disease at 13 years	Thin proximal phalanges	Thin proximal phalanges, Cervical supine anomaly
Genital Anomalies	Hypospadias, Cryptorchidism	Not observed	Hypospadias, Cryptorchidism
Puberty	Pubic hair at 11 years	Menarche at 12 years	Not observed

We obtained written informed consent for molecular studies from the patients and the parents. The Institutional Review Board of Nagoya City West Medical Center and Nagoya City University Graduate School of Medical Sciences approved this research. We also obtained written consent to publish this article from the patients and the parents.

### Exome Sequencing

Genomic DNA was isolated from the peripheral leukocytes of the siblings and parents by a standard procedure. Exome sequencing was performed with a SureSelect Human All Exon 44 Mb kit (Agilent, Santa Clara, CA) and high-throughput sequencing of pair-end reads was conducted with a HiSeq2000 system (Illumina, San Diego, CA). Data was analyzed by using a CLC Genomic Workbench 5.1 (CLC bio, Aarhus, Denmark) under the default settings.

### Sanger Sequencing

Sanger sequencing was performed by using a 3730xl DNA Analyzer (Applied Biosystems, Foster City, CA) with primer pairs 1–6 (Table 2).

**Table 2:** Nucleotides sequences of Primer Pairs.

Primer Pair	Forward Primers	Reverse Primers
1	5′-CAGGAGCCTCTCGCTGAC-3′	5′-CTTTAATGCCACGGGAGGAG-3′
2	5′-GGCGACGTAAACAAAGCTGA-3′	5′-GGGCTCTTTGGGCTCTAAAC-3′
3	5′-CGTTCCACAGGCCAAGTGCG-3′	5′-GCTGGTGCGCACTAGTACTG-3′
4	5′-CGTCCCTCCGCAGCACATCC-3′	5′-CCTGCACCGTCTCGCGGTAG-3′
5	5′-TGGACCGAAGTGGACAGCGA-3′	5′-GGGGCCAGGACCGCGACC-3′
6	5′-CGGAGCAGCTGCCTAGTGTC-3′	5′-CTTTAATGCCACGGGAGGAGG-3′
M1	5′-GCTCCGATTTCTTCGCCAAGCGCAAG-3′	5′-TCAGAGGCCCGGACAGCTTCTTGATC-3′
M2	5′-AATTTCTTCGCCAAGCGCAAGAGATC-3′	5′-GGAGATCAGAGGCCCGGACAGCTTC-3′
M3	5′-GTCGCCAAGCGCAAGAGATCAGCGCC-3′	5′-GAAATCGGAGATCAGAGGCCCGGACAGC-3′
M4	5′-AGTCGCCAAGCGCAAGAGATCAGCGCC-3′	5′-GAAATCGGAGATCAGAGGCCCGGACAGC-3′

### Plasmid Constructs and Mutagenesis

Wild-type plasmid encoding human *CDKN1C* cDNA with 3×FLAG tag at the N-terminal was constructed by insertion of a DNA fragment from pBS-human *CDKN1C* plasmid (GenBank, U22398) digested at *Sma*I and *Hind*III sites, into pCMV-3Tag-1B vector (Stratagene, La Jolla, CA) digested at *EcoR*V and *Hind*III sites. Mutant plasmids were constructed by site-directed mutagenesis with a KOD Plus Mutagenesis Kit (TOYOBO, Osaka, Japan) and primer pairs M1–4 (Table 2). Each expression plasmid was purified by using a QIAGEN Plasmid Midi Kit (QIAGEN, Hilden, Germany).

### Cell Culture and Transfection

HEK293T and HeLa cells were cultured in DMEM with 10% fetal bovine serum, 100 U/ml penicillin, and 0.1 mg/ml streptomycin. Cells were transiently transfected with expression plasmids by using Lipofectamine 2000 (Invitrogen, Carlsbad, CA) for HEK293T cells and Nucleofector 2b (Lonza, Basel, Switzerland) for HeLa cells according to the manufacturer’s instructions.

### Flow Cytometry

At 48 h after transfections, HEK293T and HeLa cells were fixed in 70% ethanol, stained by propidium iodide, and then subjected to cell cycle analysis by FACSCanto II (BD, Franklin Lakes, NJ).

### Western Blot Analysis

Cell lysates were prepared from HEK293T cells 48 h after transfection, and then immunoprecipitated with ANTI-FLAG M2 Agarose Affinity Gel (Sigma-Aldrich, St. Lois, MO). Western blot analysis was performed with input and immunoprecipitated (IP) samples by using primary antibodies against FLAG (Sigma Aldrich; F2555, 1∶1000 dilution), CDKN1C (Cell Signaling Technology, Danvers, MA; 2557S, 1∶1000 dilution), and PCNA (Abcam, Cambridge, UK; ab92729, 1∶1000 dilution). To analyze protein stability, HEK293T cells were treated with 0.1 mg/ml cycloheximide and 0.01 mg/ml MG132 (Sigma Aldrich) for 48 h.

## Results

### Identification of a Novel Mutation in the *CDKN1C* Gene

We carried out exome sequencing of the three siblings and their parents to identify a disease-causing mutation in the siblings. Pair-end reads with an average length of 90 bp were aligned to the human reference genome sequence (GRCh37/hg19), and simple nucleotide variations (SNVs) and small insertions and deletions (Indels) were called. The single nucleotide polymorphism (SNP) database dbSNP build 135 served as a reference for registered SNPs, and non-synonymous SNVs were extracted. We identified 260 variations, including homozygous and compound heterozygous variations inherited in an autosomal recessive pattern and *de novo* heterozygous variations. During the bioinformatic analysis, we were notified of the report by Arboleda et al. [9] describing mutations in *CDKN1C* in patients with IMAGe syndrome, and subsequently, we identified a non-synonymous A to C SNV with low coverage at position chr11∶2,905,905 in *CDKN1C* in the three siblings (Figure S1). This mutation was not included in the 260 candidate SNVs because it exhibited an autosomal dominant pattern of inheritance. Sanger sequencing confirmed the presence of this SNV representing c.815T>G and p.Ile272Ser (based on accession number NM_000076.2), in the PCNA-binding domain of the *CDKN1C* gene, in the three siblings and their mother (Figures S2A–C, S2E). No mutation was detected in other coding regions of the *CDKN1C* gene in the siblings. The mother was found to be an unaffected carrier, while the father was homozygous for the wild-type allele (Figure S2D).

### IMAGe-associated Mutations in the *CDKN1C* Gene did not Compromise the Protein Product Activities to Arrest the Cell Cycle

**Figure 3:**
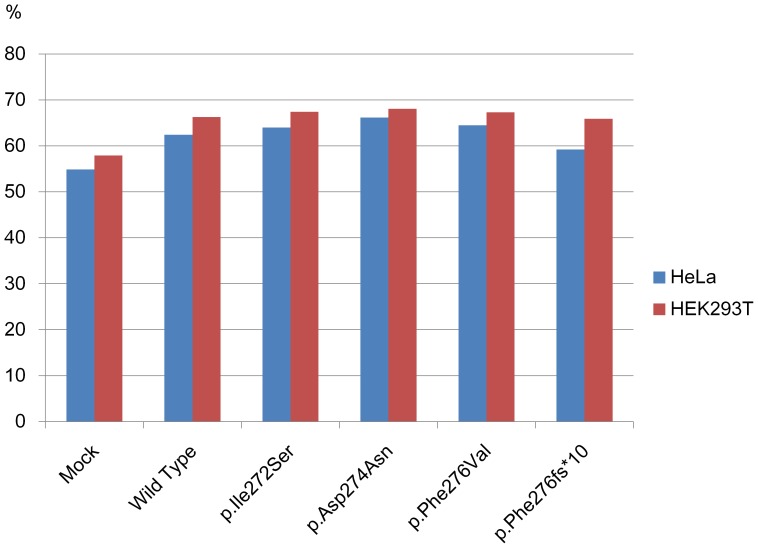
Cell cycle analysis. HeLa and HEK293T cells were transfected with plasmids expressing wild-type or one of four mutant CDKN1C proteins, p.Ile272Ser, p.Asp274Asn, p.Phe276Val, and p.Phe276fs*10. Mock transfections were performed by using pCMV-3Tag-1B vector alone. At 48 h after transfection, cells were fixed in 70% ethanol, stained by propidium iodide, and then subjected to cell cycle analysis by FACSCanto II. Percentages of the cells in G1 phase are presented by bar graphs: blue bars, HeLa cells; red bars, HEK293T cells.

We carried out a cell cycle analysis to investigate the effects of IMAGe-associated and BWS-associated mutations on cell cycle progression. HeLa and HEK293T cells were transfected with plasmids expressing wild-type and p.Ile272Ser mutant protein, as well as two known IMAGe-associated mutant proteins, p.Asp274Asn and p.Phe276Val [9], and a BWS-associated mutant protein, p.Phe276fs*10, caused by a frame-shift mutation in DNA encoding residues 276 to 285 to generate a nonsense codon at residue 286 [12]. Mock transfections were performed by using pCMV-3Tag-1B vector. FACS analysis to ascertain the proportion of cells in the various cell stages was performed 48 h later on fixed and stained cells (Figure 3). In both HeLa and HEK293T cells, the percentage of cells in the G1-phase was increased by the transfection of wild-type plasmid compared with mock transfection. Moreover, the percentages of G1-phase cells were increased further by transfection of IMAGe-associated mutant plasmids compared with wild-type plasmid, but were slightly decreased by transfection of the BWS-associated mutant plasmids. However, the differences observed did not reach significance.

### Loss of PCNA Binding in the IMAGe-associated Mutations

**Figure 4:**
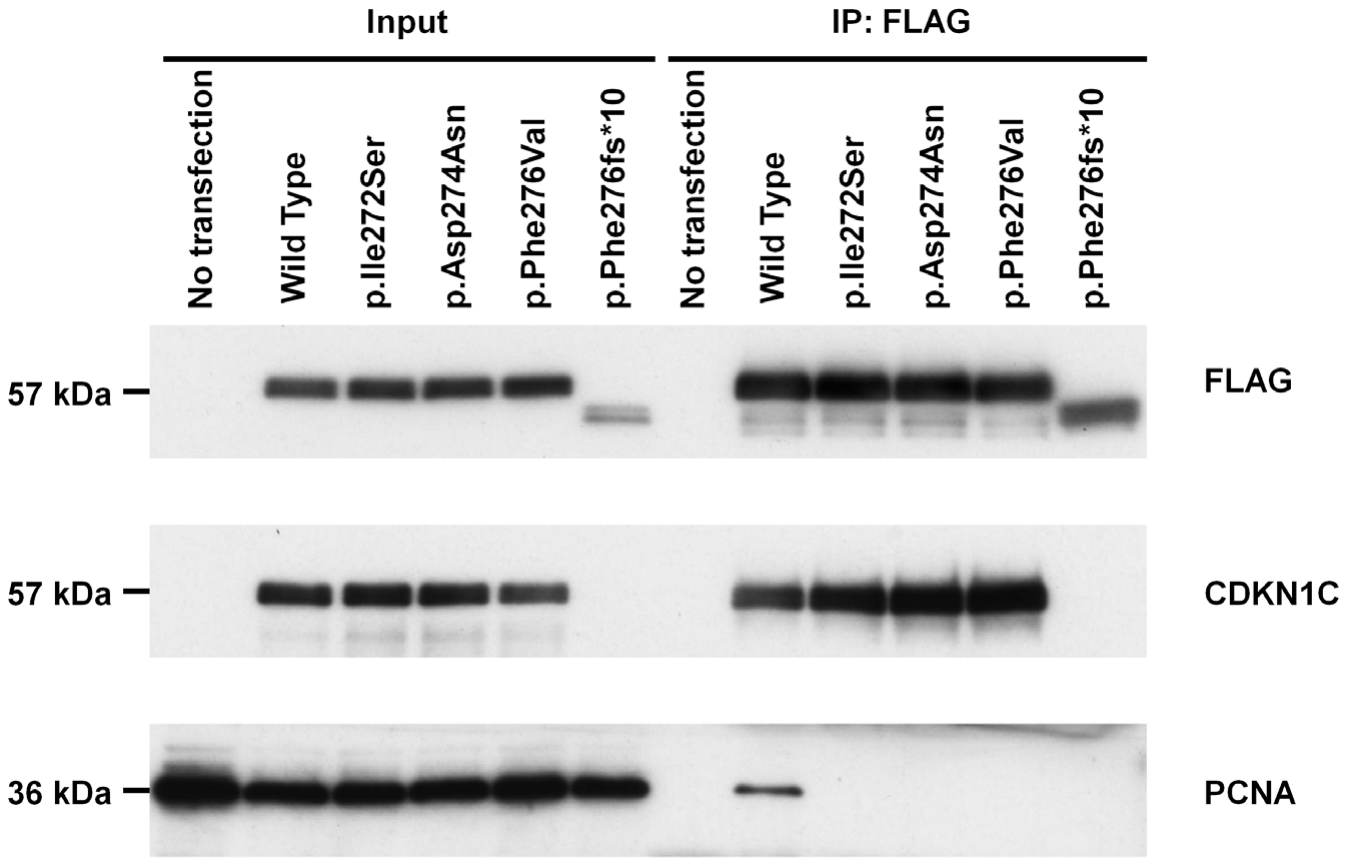
Western blot analysis. HEK293T cells were transiently transfected with plasmids expressing FLAG-tagged wild-type or one of four mutant CDKN1C proteins, p.Ile272Ser, p.Asp274Asn, p.Phe276Val, and pPhe276fs*10. Forty-eight hours after transfection, cell lysates were prepared and immunoprecipitated by anti-FLAG antibody. Both input and immunoprecipitated (IP) samples were subjected to Western blot analysis with antibodies against FLAG, CDKN1C, and PCNA.

We performed Western blot analyses to investigate the expression and PCNA-binding ability of wild-type CDKN1C protein, three IMAGe-associated mutant CDKN1C proteins, p.Ile272Ser, p.Asp274Asn and p.Phe276Val, and a BWS-associated mutant CDKN1C protein, p.Phe276fs*10. Cell lysates were prepared 48 h after transfection, and both input and IP samples were subjected to Western blot analysis by using primary antibodies against FLAG, CDKN1C, and PCNA (Figure 4). In both input and IP samples, anti-FLAG and anti-CDKN1C antibodies detected the IMAGe-associated mutant proteins, p.Ile272Ser, p.Asp274Asn, and p.Phe276Val, at the same molecular weight (∼57 kDa) and abundance as the wild-type protein. In contrast, the BWS-associated mutant protein, p.Phe276fs*10, was detected by anti-FLAG antibody at a smaller molecular weight and markedly reduced expression level compared with the wild-type protein. Moreover, the p.Phe276fs*10 mutant protein was not detected by the anti-CDKN1C antibody in the input or IP samples, indicating that it had lost immunogenicity to the antibody. Anti-PCNA antibody detected endogenous PCNA binding to wild-type CDKN1C but not to the IMAGe-associated mutant proteins, p.Ile272Ser, p.Asp274Asn, and p.Phe276Val, nor to the BWS-associated mutant protein, p.Phe276fs*10, in IP samples. We repeated the same experiments by using HeLa cells, and we confirmed the expression of wild-type and mutant CDKN1C proteins and endogenous PCNA proteins (data not shown).

### Increased Protein Stabilities in the IMAGe-associated Mutations

**Figure 5:**
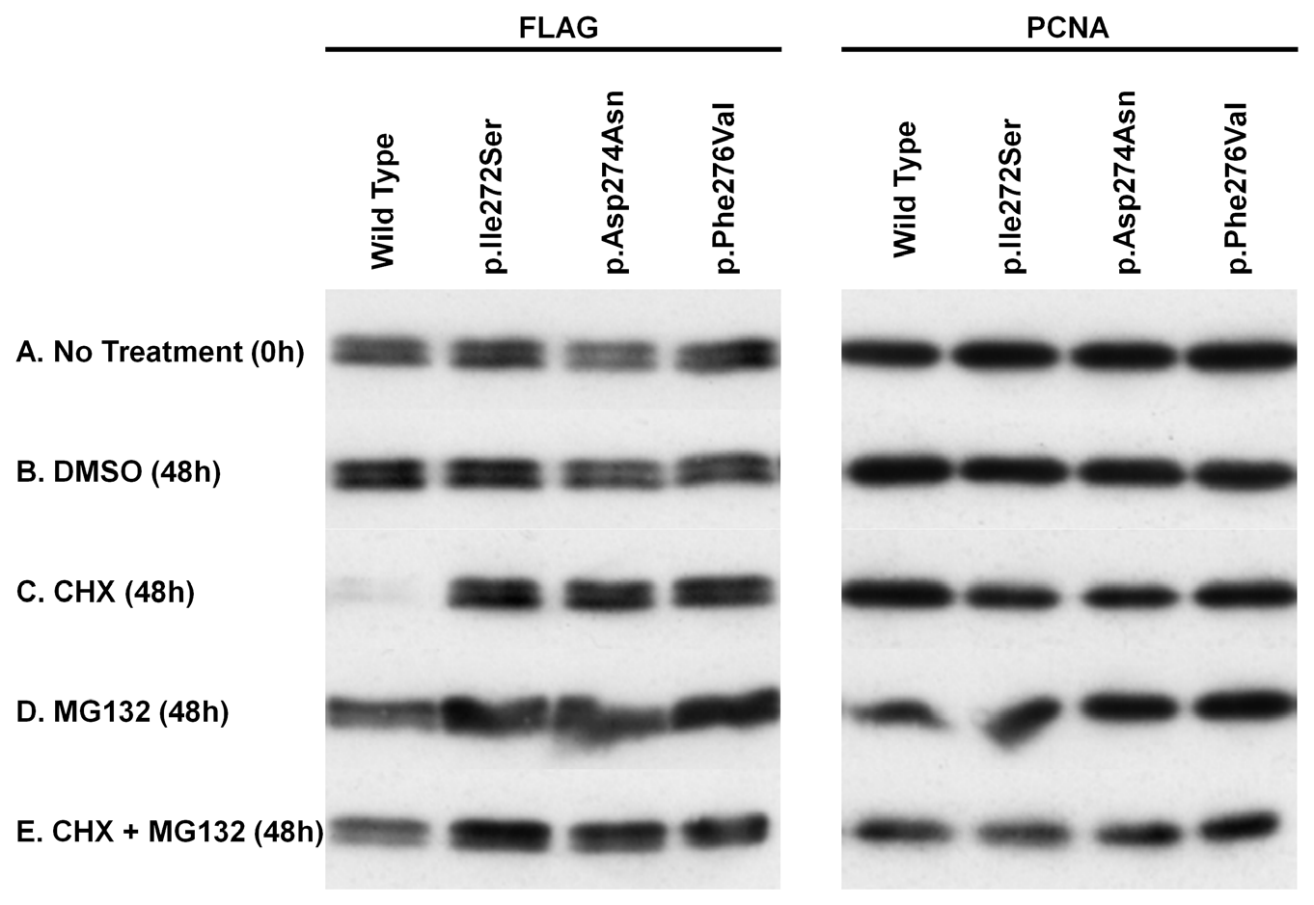
Protein stability assay. HEK293T cells were transiently transfected with plasmids expressing FLAG-tagged wild-type or one of three mutant CDKN1C proteins, p.Ile272Ser, p.Asp274Asn, and p.Phe276Val. At 48 h after transfection, cells were treated with DMSO, cycloheximide (CHX), or MG132 for 48 h. Cell lysates were prepared at 0 h (no treatment) or at 48 h (DMSO, CHX, MG132, CHX+MG132), and subjected to Western blot analysis using anti-FLAG antibody for detecting the CDKN1C proteins and anti-PCNA antibody as a loading control.

Given that a binding site for the C8 alpha-subunit of the 20S proteasome, defined in CDKN1A (Figure 1A–B), overlaps the PCNA-binding site [13], and that the motif is strongly conserved between CDKN1C and CDKN1A (Figure 1B), we analyzed the protein stability of wild-type and IMAGe-associated mutant CDKN1C proteins. HEK293T cells were transfected with wild-type and IMAGe-associated mutant plasmids (p.Ile272Ser, p.Asp274Asn, and p.Phe276Val), and the cells were treated with DMSO, cycloheximide, and MG132. Cell lysates were prepared after the treatment and analyzed by Western blotting (Figure 5). There were no differences in expression levels among wild-type and the three IMAGe-associated mutant proteins without treatment (Figure 5A) or with DMSO for 48 h (Figure 5B). In the presence of cycloheximide for 48 h, the expression levels of wild-type protein were markedly reduced, whereas IMAGe-associated mutant proteins remained the same, i.e., these proteins were remarkably stable (Figure 5C). The expression levels of wild-type proteins were also remarkably improved when treated with MG132 and cycloheximide (Figure 5E). The increased expression levels of IMAGe-associated mutant proteins were minimal when treated with MG132 alone (Figure 5D). These results indicated that the protein degradation of CDKN1C is mediated via a proteasome pathway and that the degradation is severely impaired by IMAGe-associated mutations.

## Discussion

Here we describe a novel mutation in the PCNA-binding domain of the *CDKN1C* gene in three Japanese siblings who manifest most of the IMAGe-associated symptoms: i.e., intrauterine growth restriction, adrenal hypoplasia congenita, and genital anomalies in males. Metaphyseal dysplasia, which was originally defined as one of the symptoms of IMAGe syndrome [1], was not observed in the siblings, but other bone disorders were evident. We consider that metaphyseal dysplasia might not be an essential component of IMAGe syndrome, although CDKN1C might play a role in bone disorders because *CDKN1C* knockout mice present with several bone anomalies [14]–[16]. Although over-expression of IMAGe-associated mutations in *Drosophila melanogaster* results in moderate to severe restriction in eyeball size [9], no ophthalmologic abnormalities were identified in the siblings.

The PCNA-binding motif was originally defined in CDKN1A [17], and is strongly conserved between CDKN1A and CDKN1C (Figure 1A–B). In CDKN1C, the disruption of PCNA binding partially reduces the ability of CDKN1C to suppress myc/RAS-mediated transformation [18]. In a previous study [9], p.Phe276Val and p.Lys278Glu resulted in a complete loss of PCNA binding, and p.Asp274Asn was identified in a sporadic case of IMAGe syndrome. Our findings that the amino acid changes of p.Ile272Ser, p.Asp274Asn, and p.Phe276Val resulted in the complete loss of PCNA binding indicate that Ile272 as well as Asp274 and Phe276 in humans are crucial for the ability of CDKN1C to bind PCNA (Figure 1C). However, lack of PCNA binding in CDKN1C per se is not directly involved in the gain-of-function phenotype observed in IMAGe syndrome patients because CDKN1C truncation mutants lacking PCNA binding, i.e., p.Phe276fs*10 (Figure 4), were also identified in BWS patients (Figure 1A) [11], [12], in whom the loss-of-function phenotype of CDKN1C was always observed.

The stability of Cip/Kip family CDK inhibitors is tightly regulated by ubiquitination and proteasome-mediated degradation in a manner dependent on the cell cycle stage [19], [20]. For CDKN1C, two distinct degradation complexes mediated by polyubiquitination and proteasome-associated degradation have been identified: the Skp1/Cul1/F-box (SCF)-type E3 ubiquitin ligase complex (SCF^Skp2^ complex) [21] and the TGF beta1-activated, Smad-dependent transcription of the gene encoding F-box protein (FBL12) ubiquitin ligase complex (SCF^FBL12^ complex) [22]. A Thr310 mutation in the CDKN1C protein compromised the effect of Skp2 on the degradation of CDKN1C protein, suggesting that phosphorylation at this residue is required for SCF^Skp2^ complex-mediated ubiquitination [21]. The SCF^FBL12^-mediated degradation of CDKN1C protein also requires its phosphorylation at Thr310 [22]. On the other hand, ubiquitination-independent degradation promoted by the C8 alpha-subunit of the 20S proteasome was identified in CDKN1A. Interestingly, the C8 interaction domain of CDKN1A completely overlaps the PCNA-binding site (Figure 1A–B) [13]. This degradation pathway is mediated by mouse double minute 2 (MDM2) and mouse double minute X (MDMX), both of which trigger the degradation of CDKN1A in G1 and early S phases [23], [24]. In addition, the 14-3-3 tau protein plays a role in promoting MDM2-mediated CDKN1A degradation through binding to MDM2, CDKN1A, and the C8 subunit of the 20S proteasome [25].

It is not clear whether CDKN1C is degraded by the C8 alpha-subunit of the 20S proteasome; however, it is possible that IMAGe-associated mutations disrupt binding of these two molecules because IMAGe-associated mutations are located on the putative binding site of the C8 alpha-subunit of the 20S proteasome in CDKN1C.

In conclusion, our findings clearly demonstrated that IMAGe mutations in the *CDKN1C* gene significantly stabilized the protein products. Therefore, an increase in the CDKN1C protein level can easily lead to the typical gain-of-function phenotypes observed in IMAGe syndrome patients.

## Supporting Information

Figure S1 **Mapping of exome sequencing.** Mapping results of pair-end reads around chr11∶2,905,905 (GRCh37/hg19) in patient 1 (A), patient 2 (B), and patient 3 (C) by exome sequencing are presented. The vertical red lines denote the nucleotide position on chr11∶2,905,905 (GRCh37/hg19). Genomic DNAs were isolated from peripheral leukocytes from three siblings and both parents by a standard procedure.
(TIF)

Figure S2 **Results of Sanger sequencing.** Results of Sanger sequencing to validate the A to C substitution at chr11∶2,905,905 (GRCh37/hg19) in patient 1 (A), patient 2 (B), patient 3 (C), father (D), and mother (E) are presented. A heterozygous A to C substitution at chr11∶2,905,905 representing c.815 T>G in the *CDKN1C* gene (p.Ile272Ser amino acid change) was identified in the three siblings (A, B, C) and their mother (E). The father (D) was found to be homozygous for the wild-type allele.
(TIF)
