# Supplementary figures and images for: Increased Protein Stability of CDKN1C Causes a Gain-of-Function Phenotype in Patients with IMAGe Syndrome
Source: PLoS One. 2013 Sep 30;8(9):e75137. doi: 10.1371/journal.pone.0075137 (PMC3787065; doi:10.1371/journal.pone.0075137)

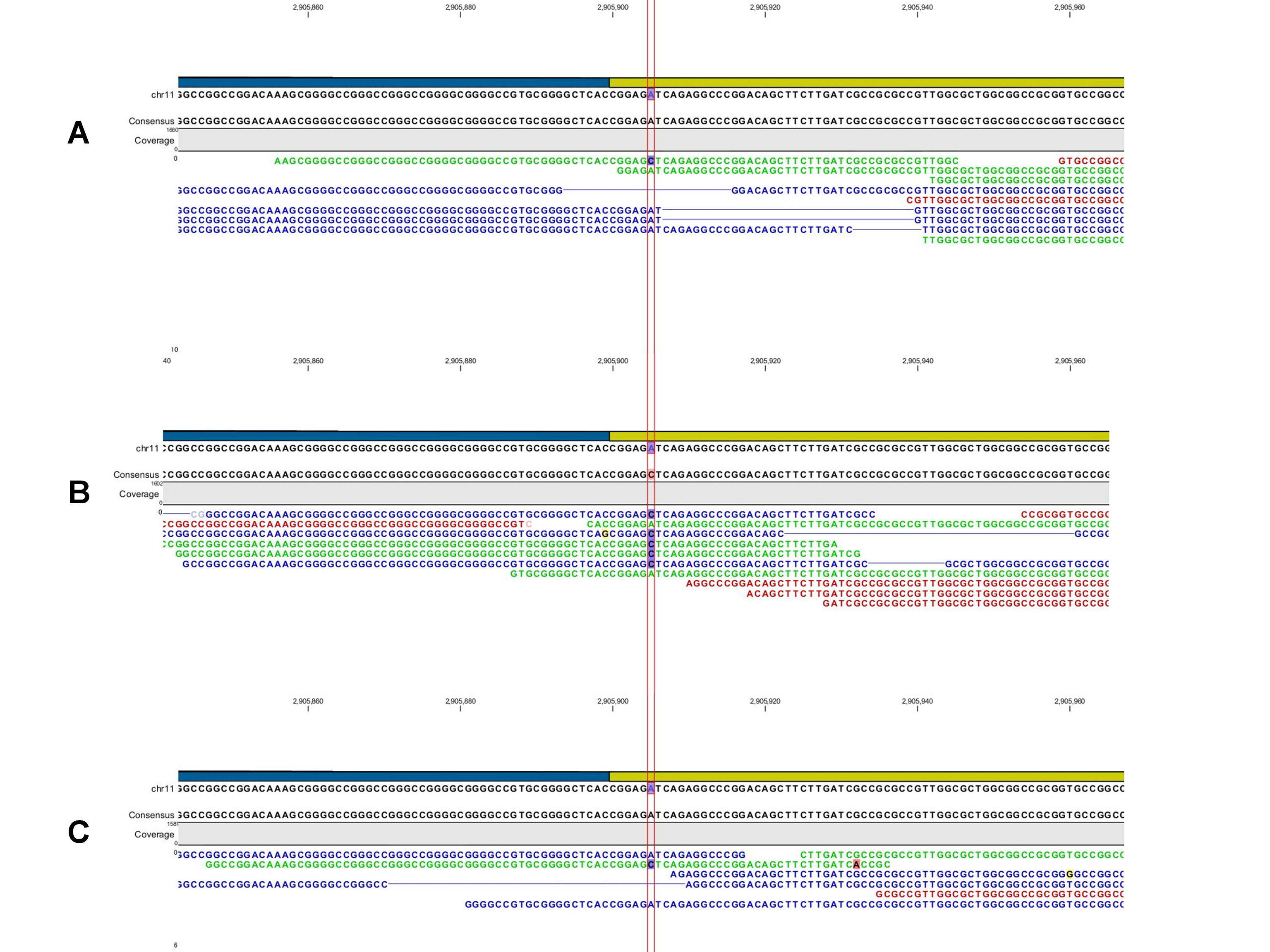

Supplement: Figure S1 — Mapping of exome sequencing. Mapping results of pair-end reads around chr11∶2,905,905 (GRCh37/hg19) in patient 1 (A), patient 2 (B), and patient 3 (C) by exome sequencing are presented. The vertical red lines denote the nucleotide position on chr11∶2,905,905 (GRCh37/hg19). Genomic DNAs were isolated from peripheral leukocytes from three siblings and both parents by a standard procedure. (TIF) [file pone.0075137.s001.tif]

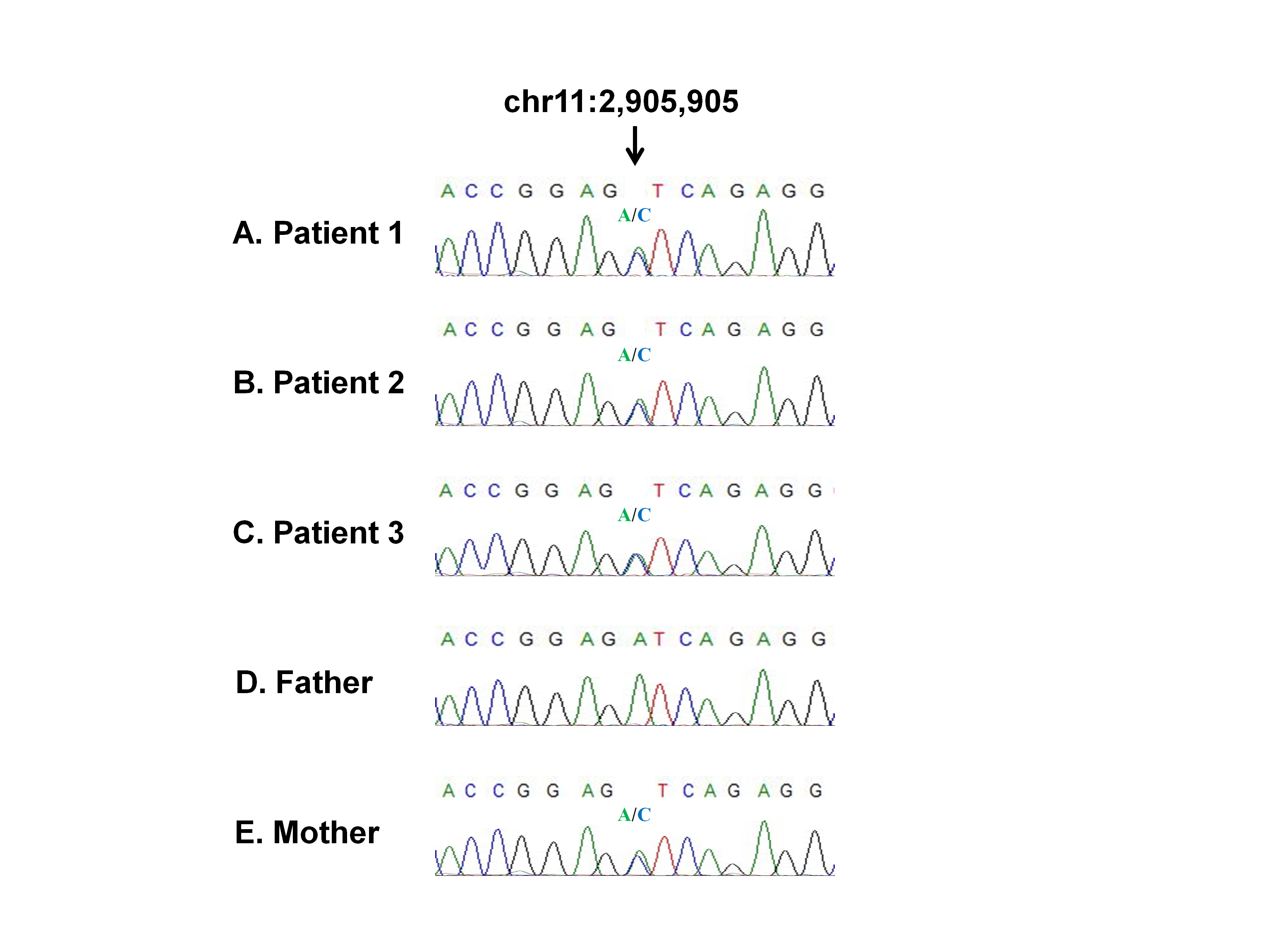

Supplement: Figure S2 — Results of Sanger sequencing. Results of Sanger sequencing to validate the A to C substitution at chr11∶2,905,905 (GRCh37/hg19) in patient 1 (A), patient 2 (B), patient 3 (C), father (D), and mother (E) are presented. A heterozygous A to C substitution at chr11∶2,905,905 representing c.815 T>G in the CDKN1C gene (p.Ile272Ser amino acid change) was identified in the three siblings (A, B, C) and their mother (E). The father (D) was found to be homozygous for the wild-type allele. (TIF) [file pone.0075137.s002.tif]
